# Beyond retrofitting: validating a purpose-built reading diagnostic assessment through a cognitive diagnostic approach

**DOI:** 10.3389/fpsyg.2026.1894358

**Published:** 2026-08-27

**Authors:** Hang Sun, Shengkai Yin

**Affiliations:** 1 College of Foreign Languages, University of Shanghai for Science and Technology, Shanghai, China; 2 Faculty of Education, Monash University, Clayton, VIC, Australia; 3 Institute of Education, Arts and Community, Federation University Australia, Berwick, VIC, Australia

**Keywords:** cognitive diagnostic approach, diagnostic assessment, EFL learners, reading assessment, test validation

## Abstract

**Introduction:**

Diagnostic assessment, which aims to identify the strengths and weaknesses of learners' cognitive abilities for targeted remediation, has gained momentum in recent years. This study applied a cognitive diagnostic approach to validate the UDig Reading Test, designed to diagnose the reading skills of tertiary-level English as a foreign language (EFL) learners in China.

**Methods:**

Using a mixed-methods design, test-takers' think-aloud protocols, expert judgments, and cognitive diagnostic modeling were integrated to examine the accuracy of the predefined item-subskill associations. Large-scale performance data from 642 test-takers were analyzed to estimate their reading subskill mastery patterns.

**Results:**

Results showed that the predefined item-subskill associations were generally supported by empirical evidence. In particular, search reading was empirically confirmed as a targeted subskill in the expeditious reading section, despite its absence from the initial test specifications. Subskill prevalence analysis identified the subskill of understanding the structure and cohesion of a passage as the most challenging one, providing evidence that may inform targeted instructional support.

**Discussion:**

This study responds to persistent calls for research into truly diagnostic assessments rather than retrofitting existing tests and demonstrates the value of the cognitive diagnostic approach in enhancing the diagnostic precision of reading assessment. It also outlines a research agenda for developing cognitive diagnostic assessment from inception so as to fully realize its diagnostic potential.

## 1 Introduction

Diagnostic assessment has attracted growing interest within the field of educational and psychological measurement in recent years. In language testing, diagnostic assessment aims to identify test-takers' strengths and weaknesses in the target language domain and provide diagnostic feedback for remedial learning (Alderson, 2005). Research shows that test-takers who achieve identical total scores may nonetheless differ in their mastery of language subskills due to individual differences (Kim, 2015). Therefore, providing test users with detailed diagnostic feedback has the potential to foster tailored teaching and learning.

Interest in diagnostic assessment is driven by increased awareness of test washback, the formative assessment movement, the application of cognitive psychology principles, and advanced psychometric techniques which support granular score reporting (Embretson and Gorin, 2001; Lee, 2015). Among these, the primary driving force is probably the rising concern about test washback, especially from large-scale, high-stakes tests which may adversely influence the teaching and learning process. Diagnostic assessment is thus conceived as an antidote because it can provide more detailed evidence of learners' ability and is learning-oriented in nature (Jang, 2008; Yin et al., 2012).

One strand of research in diagnostic assessment—cognitive diagnostic assessment (CDA)—has achieved remarkable progress during the past two decades. Conventional measurement theories including classical test theory or item response theory view proficiency as a unidimensional continuous latent trait, which are useful in positioning one examinee relative to others (de la Torre and Chiu, 2016). In contrast, the cognitive diagnostic approach conceptualizes “proficiency as a set of interrelated but separable knowledge within a domain,” thereby enabling more detailed feedback of test performance (de la Torre and Chiu, 2016, p. 253). CDA starts with the construction of a Q-matrix (Tatsuoka, 1983), an attribute-by-incidence matrix that specifies the relationships between test items and targeted cognitive attributes. Cognitive diagnostic models (CDMs) are then employed to estimate test-takers' mastery probability of each attribute (Lee and Sawaki, 2009). While CDA is a timely contribution to the field, most current research retrofits tests intended for other purposes (e.g., proficiency tests), which are limited by design in their capability to obtain adequate diagnostic information (Kim, 2015; Lee and Sawaki, 2009). As a result, there have been growing calls for research to develop and validate truly diagnostic tests.

China has a long history of examination-oriented culture and large-scale standardized testing plays a powerful role in the educational and social systems (Cheng, 2008; Qi, 2007). The test-driven culture has been shown to produce undesirable washback, often referred to as the “teaching-to-the-test” phenomenon (Tang and Biggs, 1996, p. 163). Responding to the global formative assessment movement, the Higher Education Department of the Ministry of Education in China has advocated formative assessment in the Chinese Ministry of Education (2020), aiming to leverage it as a tool to diagnose, inform, and promote learning and teaching. Accordingly, alternative assessment instruments that operationalize formative assessment are in great demand. The UDig system for higher education is developed in response to this need. The UDig is an online diagnostic test battery designed to assess the English ability of EFL learners at the tertiary level in China. The rationale for choosing this assessment system as the study instrument lies in its diagnostic design, which incorporates predetermined language subskills of interest and customized diagnostic feedback reports. This aligns with the need for research into newly developed diagnostic tests rather than retrofitting proficiency tests by statistical means (Alderson et al., 2015). With regard to the current developments as well as challenges in diagnostic assessment, this study aims to validate the UDig Reading Test by applying a cognitive diagnostic approach. By examining the validity of a diagnostic test by design, this research serves as an exploratory investigation into the theoretical and practical issues surrounding diagnostic assessment. In doing so, it not only offers empirical evidence for the validity of the UDig Reading Test, but also offers insights for future efforts in developing and validating diagnostic assessments.

## 2 Literature review

### 2.1 Theoretical principles and applications of diagnostic assessment

Although diagnostic assessment has not drawn a great deal of attention until recently, the notion of this specific test type has existed for quite some time. Early contributions to the field include speculations and discussions about the features and models of diagnostic assessment. Spolsky (1992) suggested that diagnostic assessment should be linked to curriculum and designed to provide feedback to teachers and students on progress. Shohamy (1992) proposed a cyclical diagnostic testing model which employs diverse assessment tools to provide subskill-specific feedback rather than composite scores, enabling schools to pinpoint areas to change and work toward improvement.

While these scholars have sketched an outline of diagnostic assessment, Alderson's seminal works (2005; Alderson et al., 2015) have taken the field to a new level of understanding. Alderson (2005) proposed 19 hypothetical features of diagnostic assessment. For instance, diagnostic assessment should: (1) identify strengths and weaknesses to enable targeted remediation; (2) prioritize weaknesses over strengths, and discrete lower-level language skills over integrated higher-level skills; (3) deliver immediate or minimally delayed and detailed feedback which can be acted on; and (4) operate as low- or no-stakes instruments to reduce test-taker anxiety. Alderson has given an extensive record of what diagnostic assessment is like in a way to guide its design and research, although he admitted that these features are tentative instead of definitive. Doe (2013) listed four key features to consider in developing or retrofitting tests to provide diagnostic information: content refers to whether the test draws on a general language proficiency model or a specific curriculum (Jamieson et al., 2008); grain-size defines the level of detail in diagnostic feedback (Jang, 2008); delivery specifies the method by which feedback is communicated to test users; intended use clarifies how diagnostic feedback is acted upon in teaching and learning.

In efforts to operationalize the theoretical principles of diagnostic assessment, several test batteries have been developed. DIALANG is the first well-known large-scale diagnostic assessment project. Based on can-do statements from the Common European Framework of Reference for Languages (CEFR), it comprises diagnosis of five language skills across 14 European languages. Users complete a vocabulary size placement test (VSPT) and a self-assessment before being presented with items calibrated to easy, medium, or difficult levels. After taking the test, users receive immediate feedback, including correct answers, their responses, the target skills, and VSPT and self-assessment results. DIALANG serves as a robust attempt to put the idea of diagnostic assessment into practice. However, it has been criticized since too few target skills are assessed to be considered fine-grained and the feedback is too general to guide test-takers for further actions (Alderson et al., 2015). To assess international students' readiness for study at an English-medium university, Diagnostic English Language Needs Assessment (DELNA) has been introduced at the University of Auckland. In the DELNA programme, all first-year undergraduate students complete a 2-h diagnosis including reading, writing, and listening tasks after a 30-min screening test (vocabulary and timed reading). DELNA report follows a 6-point summary band scale which describes what typical students can do at each level. However, as DELNA only reports students' skill level in terms of general bands, rather than granular feedback, it is more appropriate to be referred to as a post-entry test (Dunworth, 2009; Read, 2013). Another diagnostic assessment project is Diagnostic English Language Tracking Assessment (DELTA), developed by Hong Kong Polytechnic University and partner institutions. The test consists of vocabulary, grammar, listening, and reading sections. Students receive individualized component skills profiles after the test. Additionally, DELTA provides follow-up learning resources, including guidance from teachers or advisors and learning materials for independent study (Urmston et al., 2013).

Taken as a whole, these tests all fall under the broad umbrella of diagnostic assessment, although they approach the concept differently. For instance, they appear to assess different aspects of language skills at different levels. In reading diagnosis, DIALANG measures three reading subskills including “understanding the main idea” and “understanding specific details”; DELNA measures eight skills, such as “locating causes and effects, sequences, contrasts” and “selecting words which fit the meaning and the grammatical construction of the text”; DELTA, by contrast, assesses reading subskills across various text types (e.g., news articles and fiction) and themes (e.g., history and culture). These tests also differ in the way feedback is delivered and structured. To sum up, there are some promising projects underway, but more work still needs to be done to advance the theoretical basis and practical applications of diagnostic assessment.

### 2.2 Current developments in cognitive diagnostic assessment

CDA intends to assess test-takers' specific knowledge structures to generate fine-grained diagnostic feedback. Progress in CDA can be attributed to the advancements in psychological measurement and insights from cognitive psychology. As Leighton et al. (2007) contended, researchers are finally in a position to design assessments capable of providing detailed insights into students' cognitive strengths and weaknesses.

CDA usually involves three steps. First, targeted attributes are identified. In CDA, attributes refer to specific skills, knowledge, and abilities necessary for a correct response to a test item (Buck and Tatsuoka, 1998). A Q-matrix, which is an incidence matrix that specifies how test items align with particular attributes (Tatsuoka, 1983), should be carefully constructed. In a binary *J* × *K* Q-matrix, *J* represents the number of test items and *K* the number of attributes. Each entry *q*_*jk*_ is coded 1 if solving item *j* involves attribute *k*, and 0 otherwise. Accuracy of the Q-matrix is of paramount importance for it governs the precision of diagnostic outcomes (Rupp and Templin, 2008). Second, attribute mastery patterns are obtained by applying CDMs to analyze examinees' test response. More than 60 CDMs have been developed (Fu and Li, 2007) and studies have compared the CDMs and sought ways to select the most suitable models under different conditions (e.g., Gao et al., 2018; Tu et al., 2013). Of the many variables characterizing CDMs, two distinctive features are usually taken into consideration when selecting a model: whether the model is conjunctive or disjunctive, and whether it is saturated or reduced. Conjunctive or non-compensatory models require examinees to possess all necessary attributes to answer an item correctly. Rule Space Model (RSM; Tatsuoka, 1983) and Deterministic Input, Noisy “and” Gate (DINA; Junker and Sijtsma, 2001) are conjunctive models. In contrast, disjunctive or compensatory models posit that strengths in some attributes can offset the absence of another. A representative example is Deterministic Input, Noise “or” Gate (DINO; Templin and Henson, 2006). In cases where attribute relationships are unclear, a saturated or unconstrained model is preferred as it offers greater flexibility than reduced or constrained model (Li et al., 2016). Some well-known saturated models include General Diagnostic Model (GDM; von Davier, 2005) and Generalized Deterministic Inputs, Noisy “and” Gate model (G-DINA; de la Torre, 2011). However, saturated models generally demand a larger sample for stable estimation, due to their complexity (Li et al., 2016). Thus, the selection of an appropriate CDM should depend on both theoretical considerations of the underlying construct and empirical evidence of model-data fit. Once an appropriate model has been selected, it is calibrated to generate information such as attribute prevalence, proportions of attribute patterns, and individual examinee's attribute mastery probabilities. At the final step, detailed diagnostic feedback should be carefully designed and communicated to stakeholders.

A handful of studies have explored the application of the cognitive diagnostic approach in L2 assessment. Jang (2005) made a substantive contribution to the field by retrofitting the reading section of *LanguEdge* courseware, a prototype of the TOEFL, to generate diagnostic information. She explored how the Fusion Model generated skill profiles and further examined the pedagogical impact of diagnostic feedback in two TOEFL preparation courses. Chen and Chen (2016) validated the G-DINA model's application in the PISA reading test, finding that the compensatory and saturated model accommodated the multi-dimensionality of language tests and the interrelated relationships among the reading attributes. In similar fashion, Mirzaei et al. (2020) employed the G-DINA model to retrofit the IELTS reading section to analyze the overall and individual reading performance of Iranian students, as well as the differences in attribute mastery between those in engineering and veterinary fields. These studies apply *ex post facto* CDA to extract diagnostic information from non-diagnostic large-scale tests. A fundamental problem with this approach is that there are often not enough items addressing specific attributes (Kim, 2015; Lee and Sawaki, 2009). As a result, some initial attributes, though important, are dropped from the Q-matrix to fit the statistical analysis. To date, research on diagnostic assessment designed *a priori* remains scarce, with notable exceptions including Kim (2010) and Toprak and Cakir (2021). In Kim's (2010) study, the Reduced Reparameterized Unified Model was applied to demonstrate the diagnostic power of an empirically-derived, descriptor-based checklist for assessing academic English writing ability. Toprak and Cakir (2021) designed a diagnostic reading test and applied the log-linear cognitive diagnosis model to examine adult learners' reading ability in a Turkish EFL academic setting. These studies stand as pioneering additions to the CDA literature.

### 2.3 Assessing second language (L2) reading

Unlike assessing productive skills, assessing receptive skills such as reading is more complicated because the actual reading process occurs privately within the reader and cannot be directly observed or investigated (Alderson et al., 2015). In effect, how we assess reading ability hinges on how we conceptualize and operationalize the constructs of reading.

Enright et al. (2000) presented three perspectives in conceptualizing the constructs of reading comprehension for the TOEFL 2000 project—the processing perspective, the task perspective, and the reader purpose perspective. The processing approach views reading as driven by various linguistic and processing variables, including lower-level skills, such as word recognition, and higher-level comprehension abilities like inferencing. The second perspective uses task-based variables to define reading construct. In that way, the construct of reading is operationalized into authentic tasks that take place in the real world. The reader purpose perspective, which was employed by the TOEFL 2000 project, examines the differing purposes of reading. Enright et al. (2000, p. 1) listed four academic reading purposes: “reading to find information,” “reading for basic comprehension,” “reading to learn,” and “reading to integrate information across multiple texts.” Notably, distinctions among these perspectives are not always clear-cut. For instance, “reading to integrate information across multiple texts” as a reader's purpose can also be categorized as a processing skill “synthesizing and summarizing main ideas.” Also, the choice of perspective depends on the test contexts and purposes. For example, the task-based perspective is more widely used in language tests designed for specific purposes such as occupational purposes, when test-takers are required to accomplish specific tasks in the target domain.

Khalifa and Weir (2009) proposed a cognitive processing approach to define the construct measured by reading tests and examine tests' cognitive validity. Khalifa and Weir's model emphasized the interplay among readers' goal-setting, cognitive processing and knowledge base. Readers' decision of what type of reading to employ determines which levels of processing are engaged in the model's central core. Reading goals can be either expeditious or careful, and may take place at the local or global level. The processing core includes eight cognitive processes in a hierarchical system with the lower levels comprised of word recognition, lexical access, syntactic parsing, establishing propositional meaning, and the higher levels including inferencing, building a mental model, and creating a text level or intertextual representation (Khalifa and Weir, 2009). Lower-level cognitive processes refer to local comprehension (understanding of propositions within the sentence), while the higher levels involve global comprehension beyond the sentence level (Weir et al., 2009). The knowledge base readers deploy when engaged in reading is linked to specific levels of the processing core. As Jin (1998) put it, because of practical pedagogical and testing needs, research in L2 reading tends to take a product-oriented approach to investigating the nature of reading, with relatively little focus on the cognitive processing *per se*. The merit of Khalifa and Weir's model is that it highlights the central role of test-takers and brings to light test-takers' underlying cognitive processes that govern their reading activities.

Turning to the UDig Reading Test, to align with L2 reading comprehension models and fit into the Chinese higher education context, its design incorporates both theoretical frameworks, such as Khalifa and Weir's (2009) model, and local college English curriculum requirements and test specifications from established tests like the College English Test (CET). The targeted reading subskills in the UDig test are conceptualized and operationalized to encompass both expeditious reading skills and careful reading skills, as well as lower-level skills and higher-level skills. Further details on these subskills are provided in the Methods Section.

### 2.4 Research gap and the present study

As evidenced by the growing literature, CDA has made considerable inroads into the field of language assessment, with empirical studies showing the practicality of the CDMs in extracting diagnostic information. However, existing research largely relies on retrofitting non-diagnostic tests, resulting in both methodological and theoretical limitations. Firstly, this approach faces psychometric challenges such as unbalanced item distributions, flat mastery profiles, and model fit issues (Lee and Sawaki, 2009; Rupp and Templin, 2008), which jeopardize the richness and robustness of diagnosis. Secondly, theoretical problems arise with the *post hoc* identification of attributes, as it fails to align with cognitive models of reading. Due to the lack of access to explicit test specifications or underlying theoretical frameworks, retrofitting research often turns to certain taxonomies for attribute definition and item-by-attribute relationships are usually inferred afterward. Additionally, without featuring a diagnostic function at the point of test construction, the coverage of reading subskills is somewhat constrained. For instance, expeditious reading subskills and certain higher-level subskills, although critical in L2 reading, are seldom included in the diagnosis. On the other hand, conventional diagnostic assessments like the UDig face the limitation that one item is designated to only one subskill, while many test items appear to tap more than one subskill based on substantive and empirical grounds (Yi, 2016; Chen et al., 2023). Addressing these gaps, this study explores how a cognitive diagnostic approach can be applied to validate a conventional diagnostic assessment, namely the UDig Reading Test. As diagnostic assessment intends to generate fine-grained mastery profiles of test-takers' ability, establishing evidence that the test actually measures the intended constructs is of utmost importance (Sun, 2020). This study thus seeks to answer the following questions:

1. To what extent are the item-by-subskill relationships validated by the cognitive diagnostic approach consistent with the test specifications?
2. What are the characteristics of UDig test-takers' reading subskill mastery patterns, as revealed by the cognitive diagnostic approach?

## 3 Methods

### 3.1 Research design

This study employed an exploratory mixed-methods design (Creswell and Clark, 2017), in which qualitative data collection and analysis preceded and informed quantitative phases. Since test developers' intentions are not sufficient to guarantee that test items tap into the targeted reading subskills, test-takers' verbal reports, expert judgments, and cognitive diagnostic modeling were employed collectively to examine item-by-subskill relationships. First, students' verbal reports and expert judgments were collected to generate qualitative descriptions of the subskills measured by test items. A provisional Q-matrix was derived from these qualitative results and subsequently contrasted with the initial specification-based Q-matrix. Second, the better fitting Q-matrix was modified by the cognitive diagnostic approach. Lastly, based on the refined Q-matrix and test-takers' response data, quantitative statistical modeling was used to validate the diagnostic psychometric properties and generate information about test-takers' mastery of reading subskills.

### 3.2 Participants

This study recruited three groups of participants for think-aloud protocols, expert judgments, and cognitive diagnostic modeling. The first group of think-aloud protocol participants comprised eight college students (three females and five males; aged 19 to 21 years) from two Chinese universities, majoring in different disciplines. The second group included six college EFL teachers (five females and one male) with extensive experience in teaching reading. All of them held a PhD or master's degree in Applied Linguistics or English Language and Literature. Their average teaching years were about 11 years. These teachers completed a judgment survey to identify the relationship between the targeted subskills and test items. The third group of UDig field test participants comprised 642 students from two Chinese universities. The test-takers were gender-balanced, majored in different disciplines, and were in their second year of undergraduate study, approximating the general population of UDig level 5.

### 3.3 Instruments

#### 3.3.1 The UDig level 5 reading test

The UDig assessment system consists of six modules (reading, listening, speaking, writing, translation, and vocabulary and grammar) and four proficiency levels (UDig 4–7). Test instrument used in this study was a prototype of the UDig Level 5 Reading Test, targeting second-year non-English major students. The reading diagnosis is divided into two timed sections: expeditious reading (1 passage, 15 min) and careful reading (5 passages, 45 min). The expeditious reading section includes two item types (true or false and multiple-choice), designed to assess the subskills of “scan to locate relevant information” and “skim to identify main ideas,” respectively. The careful reading section contains four task types (multiple-choice, summary completion, cross-text multiple matching, and gapped text), targeting the subskills of “identify specific details,” “identify main ideas and significant points,” “make inferences,” “compare viewpoints,” and “understand the structure and cohesion of a passage.” Seven targeted reading subskills and their descriptors in the UDig test specifications are presented in Table 1. The test includes 32 items. Notably, Item 10 in the expeditious reading section—the only item measuring “skim to identify main ideas”—was not reported in the UDig diagnostic report. Consequently, the original subskill “skim to identify main ideas” was not evaluated in the follow-up cognitive diagnostic analyses. Instead, think-aloud protocols and expert judgments supported the inclusion of “search reading” as an additional targeted subskill during Q matrix validation. After test completion, the system automatically generates individualized diagnostic reports for students, providing information of their overall performance, subskills-specific performance, and advice for future improvement. Whole-class diagnostic reports are also available for teachers.

**Table 1:** UDig reading subskills and descriptors.

Subskills	Descriptors
Scan to locate relevant information	Can quickly identify the location of given information when reading relatively long and easy texts
Skim to identify main ideas	Can quickly obtain the main ideas when reading relatively long and easy texts
Identify specific details	Can find and understand detailed information, such as time, location, and concept
Identify main ideas and significant points	Can summarize the main ideas of the text or the paragraphs Can understand significant points that are explicitly stated and relevant to the main ideas
Make inferences	Can infer the meaning of a new word from the context Can infer the meaning of information implicitly stated Can infer the author's intention, opinion or attitude
Understand the structure and cohesion of a passage	Can understand the structure of the text and the logical relationships at the sentence and paragraph levels
Compare viewpoints	Can compare and analyze different viewpoints and attitudes when reading texts on the same topic

#### 3.3.2 Think-aloud protocol

A think-aloud protocol instrument was designed to elicit test-takers' reading subskills. This study employed the concurrent introspective approach, as it avoids the memory limitations and reconstructive biases often present in retrospective reports (Cohen and Upton, 2006). The instrument consisted of participants' background information, task instructions, training tasks, sample facilitating questions, and following-up interview questions.

#### 3.3.3 Expert judgment survey

An expert judgment survey was developed and distributed to six expert teachers. The first part of the survey collected participants' background information, while the second part provided detailed instructions for rating. Definitions and descriptions of the reading subskills were presented both in English and Chinese. In addition to the seven subskills outlined in the test specifications, an additional subskill—“search reading to extract information on a predetermined topic”—was added to the rating form. This subskill was identified as critical for expeditious reading both through students' think-aloud protocols and in prior research. A separate column allowed teachers to note any subskills not included in the list.

### 3.4 Data collection and analysis

#### 3.4.1 Think-aloud protocol

The verbal protocol was conducted in a quiet and secure environment. A training session, starting with an explanation of the purpose and principles of the think-aloud activity was held prior to the formal procedure. Two practice tasks—an anagram and a short passage reading comprehension from Cohen and Upton (2006)—were conducted to familiarize participants with the process. Each student was assigned three out of six reading passages. Participants could verbalize their thoughts in Chinese or English. To minimize interruptions, only when participants remained silent for more than a few second, did the researcher prompt them with cues. Follow-up interviews were conducted after each session to clarify unclear statements. The think-aloud protocols were recorded and transcribed shortly after the sessions. Drawing on literature on reading strategies (e.g., Cohen and Upton, 2006; Jang, 2005; Khalifa and Weir, 2009), a coding scheme of 15 reading subskills was initially developed. To avoid potential bias, a second coder independently coded four think-aloud protocols. The inter-coder agreement rate was 81.4%, indicating that the transcriptions were consistently coded.

#### 3.4.2 Expert judgments on the subskills

Six expert teachers were asked to review the passages and specify the reading subskills measured by each test item. They completed the task individually, identifying one or more subskills per item and classifying each subskill as primary or secondary according to its relative importance in solving the question. The identified subskills were then carefully reviewed. Two types of reading subskills received particular attention: those eliciting high disagreements among experts and between experts and the test developer. Results from expert judgments were cross-referenced with students' verbal protocols to form a more comprehensive view of the targeted subskills.

#### 3.4.3 Statistical diagnosis modeling

Responses from 642 test-takers on 31 test items (item 10 removed) were submitted for cognitive diagnostic modeling in R, “GDINA” package, version 2.7.3 (Ma and de la Torre, 2019a). The first stage involved examining the appropriateness of the Q-matrices, including an initial Q-matrix based on the UDig test developer's intention and a provisional Q-matrix informed by test-takers' verbal reports and expert judgments. In cases where the true model is uncertain, a saturated model can serve as the true model to evaluate Q-matrix misspecification (Chen et al., 2013). The G-DINA model, a widely researched and validated saturated model, was employed in this case. The G-DINA model is “an interpretable model based on the identity link function and represents one of the many alternative general CDM formulations” (de la Torre, 2011, p. 196). As shown in the following equation (de la Torre, 2011), the probability of a correct response on item *j* is expressed as the sum of an intercept, the main effects of the required attributes, and their interactions. The intercept δ_j0_ denotes baseline success and δ_jk_ is the contribution of mastering α_k_. $\alpha_{\text{k}}.{\text{δ}}_{\text{jkk}},$ captures the interaction effect due to α_k_ and $\alpha_{\text{k}}$, and $\delta_{\text{j}12\cdot \cdot \cdot K_{\text{j}}^{*}}$ is the joint interaction of all required attributes. Absolute fit statistics, including the maximum *z*-scores for three indices—the proportion correct (*p*), transformed correlation (*r*), and log-odds ratio (*l*) (de la Torre and Douglas, 2008; Sinharay and Almond, 2007)—were used to detect possible Q-matrix misspecifications. With *J* items, there are *J* item-level residuals and *J*(*J*−1)/2 pairwise residuals. Thus, the maximum *z*-score of each statistic was examined, which obviates the need to test each item and item-pair (Chen et al., 2013; Chen and Chen, 2016). Holm-adjusted significance levels were applied to control Type I error.


$$\begin{array}{l} P\left(\alpha_{lj^{*}}\right)=\delta_{j0}+\sum_{k=1}^{K_{j}^{*}}\delta_{jk}\alpha_{lk}+\sum_{k=1}^{K_{j}^{*}-1}\sum_{k^{'}=k+1}^{K_{j}^{*}}\delta_{jk}\alpha_{lk}^{'}\alpha_{lk}^{'}\\ \;+\cdots +\delta_{j12\cdots K_{j}^{*}}\prod_{k=1}^{K_{j}^{*}}\alpha_{lk} \end{array}$$

At the second stage, the Q-matrix which manifested a good model fit was further validated using the stepwise Wald test method (Ma and de la Torre, 2019b). (de la Torre and Chiu 2016) introduced a discrimination index $\varsigma_{\text{j}}^{2}$ for detecting Q-matrix misspecifications by identifying q-vectors that maximize variance in success probabilities across different latent groups (Nájera et al., 2019). However, specifying all attributes in a q-vector inflates $\varsigma_{\text{j}}^{2}$ as additional attributes generate more latent groups, heightening the variability in success probabilities (Nájera et al., 2019). To address this, the proportion of variance accounted for (PVAF) is adopted under the parsimony principle as a stopping rule (de la Torre and Chiu, 2016). (Ma and de la Torre 2019b) extended this approach with the stepwise Wald test, which statistically evaluates whether removing an attribute significantly worsens model-data fit. The procedure begins by identifying the first attribute with PVAF and then iteratively adds other attributes, guided by both PVAF and the Wald test (Ma and de la Torre, 2019b). The chosen Q-matrix from stage one was validated and the modifications suggested were examined. A final Q-matrix was constructed, which was then compared with the initial Q-matrix to examine the extent to which the test measured the subskills it intended to measure.

At the third stage, relative fit statistics were computed for competing models to see if any reduced CDMs could provide a better data fit than the saturated model. Relative fit evaluations included −2 log likelihood (-2LL), the conventional Akaike's information criterion (AIC; Akaike, 1974) and Bayesian information criterion (BIC; Schwarz, 1978). The model yielding the lowest value of these statistics was chosen. Posterior probabilities of test-takers' attribute mastery were established based on the input of the final Q-matrix and the best-fitting model.

## 4 Results

### 4.1 Item-by-subskill relationships

#### 4.1.1 Students' think-aloud protocols

As mentioned in the Methods section, 15 reading subskills were coded in the think-aloud protocols. However, the primary processing subskills for diagnostic purposes should be distinguishable, testable, and measured by a sufficient number of items. Accordingly, a few related subskills were clustered into broader categories and those not elicited by the tasks or with minimal influence on successful task completion were excluded. This yielded eight primary subskills, including the seven subskills in the test specifications and an additional one—search reading. In general, students' verbal reports largely supported the test specifications, though a few discrepancies were observed.

Firstly, the strategy of search reading, despite its absence from the test specifications, was observed to be required in the expeditious reading section. Scanning refers to selective reading aimed at locating very specific information, such as particular words, names, and dates (Khalifa and Weir, 2009). Search reading involves sampling text elements like words, topic sentences, or key paragraphs to search for pertinent information, with the predetermined theme in mind (Jin, 1998; Urquhart and Weir, 1998). The following example, which required students to make a judgment about a statement, manifests why the item actually required the strategy of search reading, rather than scanning:

The statement: The Faiyum, an oasis to the west of the Nile River, is formed by springs.

Relevant information in the text: The Faiyum (also spelled Fayum and Fayyum) lies in a kind of natural bowl or depression to the west of the Nile River…Oases formed by springs are visible because they are spots filled with plants that end abruptly on all sides as they give way to dry desert. The Faiyum, however, is slightly different. It is formed by channeled water—in this case, a channel that branches off from the Nile.

Excerpt from Student 5: “Springs,” key word, “springs.” (Repeats 5 times). Look for it. “However,” this thing, “springs,” eh… where is it? “Springs.” It should have this key word. Let me look for the preceding part, this. (Reads some of the words from the paragraph). “The Faiyum lies in a kind of natural bowl or depression to the west of the Nile River.” This is right. And then it is this thing…no problem. And whether it is formed by this spring. (Reads some of the words from the paragraph). “Oases formed by spring.” Eh, it should be this. It has all the key words.

*(Note: Quotes from students were translated from Chinese, and the components in the quotation marks were original English produced by the students)*.

The statement focused on Faiyum's location and formation. The location of the Faiyum could be quickly identified through simple word matching. In terms of the formation, the text explained the characteristics of oases formed by springs and then went on to illustrate the distinctiveness of Faiyum as formed by channeled water. As shown in the excerpt, Student 5 used keyword scanning to verify information, but failed to notice the key contrast in the argument transition. Consequently, he incorrectly judged the statement true. However, the word matching alone was not adequate in fulfilling the goal in this case.

Secondly, a discrepancy was found between the expected subskill of item 17—identify main idea and significant points—and the subskill revealed from the verbalizations—identify specific details. The question stated that “Mahua literature is essential because (blank).” The correct answer is “it makes it possible for Chinese writers to connect with writers from other ethnic groups,” which is a paraphrase of the sentence in the passage “It is also important in building a bridge of understanding between Chinese writers and writers of other ethnic groups in our country through translations.” Student 2 also reported that she selected the right answer because the expression of the key option was almost the same as a sentence in the text. Thus, this item was more appropriate to be coded as measuring the strategy of identifying specific details.

Thirdly, test-takers were observed to employ more than one subskill in solving some of the tasks. For instance, the subskill of comparing viewpoints was specified in cross-text multiple matching tasks, in which test-takers needed to read four texts on the same topic and choose a text to match a prompt. In conjunction with this subskill, the subskill of identifying main ideas and significant points also served as basis. These findings were further verified through expert judgments to collaboratively refine item-by-skill relationships for cognitive diagnostic modeling.

#### 4.1.2 Expert judgments

Six experts made judgments about the primary as well as secondary subskills required by each item. Twenty-six out of thirty-one items (84%) had an agreement rate of 60% or above on the primary subskills. In general, test developer's specifications were confirmed to a large extent. Also, experts identified no subskills other than the proposed ones.

A closer inspection of the judgments revealed several major points. Firstly, it was clear that experts' ratings confirmed the requirement of search reading strategy for a number of items. However, experts demonstrated lower consistency in distinguishing between scanning and search reading. In this vein, students' verbalizations and item content analysis served as reference in specifying the targeted subskills. Secondly, none of the experts considered item 17 to be targeting the subskill of identifying main ideas and significant points, which again confirmed the results from the think-aloud protocols. However, experts were divided on this item, with some supporting the subskill of making inferences, others voting for identifying specific details. Thirdly, raters identified secondary subskills for several items. Tasks with high frequencies of requiring secondary subskills were multiple-text matching and gapped-text tasks, which mirrored the findings from students' think-aloud protocols. In terms of the relative importance of the subskills in responding to test items, expert judgments confirmed the subskills proposed by the test developer were the primary ones.

#### 4.1.3 Construction of the initial and provisional Q-matrices

Based on the test developer's intention, an initial item-by-attribute Q-matrix (Q1) was constructed, with one item matched to only one attribute. In comparison, a provisional Q-matrix (Q2) was developed based on results of students' think-aloud protocols and expert judgments. An examination of Table 2 reveals the differences between Q1 and Q2. First, search reading (A2) was included in the expeditious reading section (items 1 to 9) in Q2 and items 2, 3, 5, 7 were assigned to this subskill. Second, secondary subskills, including A3 (Details), A4 (Main ideas), and A5 (Inferences) were assigned to items 13, 14, 23-32, 20. Third, the attribute assigned to item 17 was rectified to A3 (Details) in Q2 from A4 (Main ideas) in Q1.

**Table 2:** Initial and provisional Q-matrices.

Item	Q1						Q2						
	A1	A3	A4	A5	A6	A7	A1	A2	A3	A4	A5	A6	A7
1	1	0	0	0	0	0	1	0	0	0	0	0	0
2	1	0	0	0	0	0	0	1	0	0	0	0	0
3	1	0	0	0	0	0	0	1	0	0	0	0	0
4	1	0	0	0	0	0	1	0	0	0	0	0	0
5	1	0	0	0	0	0	0	1	0	0	0	0	0
6	1	0	0	0	0	0	1	0	0	0	0	0	0
7	1	0	0	0	0	0	0	1	0	0	0	0	0
8	1	0	0	0	0	0	1	0	0	0	0	0	0
9	1	0	0	0	0	0	1	0	0	0	0	0	0
11	0	1	0	0	0	0	0	0	1	0	0	0	0
12	0	0	0	1	0	0	0	0	0	0	1	0	0
13	0	0	0	1	0	0	0	0	1	0	1	0	0
14	0	0	1	0	0	0	0	0	1	1	0	0	0
15	0	1	0	0	0	0	0	0	1	0	0	0	0
16	0	0	1	0	0	0	0	0	0	1	0	0	0
17	0	0	1	0	0	0	0	0	1	0	0	0	0
18	0	0	0	1	0	0	0	0	0	0	1	0	0
19	0	1	0	0	0	0	0	0	1	0	0	0	0
20	0	1	0	0	0	0	0	0	1	0	1	0	0
21	0	0	0	1	0	0	0	0	0	0	1	0	0
22	0	0	1	0	0	0	0	0	0	1	0	0	0
23	0	0	0	0	0	1	0	0	0	1	0	0	1
24	0	0	0	0	0	1	0	0	0	1	0	0	1
25	0	0	0	0	0	1	0	0	0	1	0	0	1
26	0	0	0	0	0	1	0	0	0	1	0	0	1
27	0	0	0	0	0	1	0	0	0	1	0	0	1
28	0	0	0	0	1	0	0	0	0	1	0	1	0
29	0	0	0	0	1	0	0	0	0	1	0	1	0
30	0	0	0	0	1	0	0	0	0	1	0	1	0
31	0	0	0	0	1	0	0	0	0	1	0	1	0
32	0	0	0	0	1	0	0	0	0	1	0	1	0

(1) A1: scan to locate relevant information (Scanning), (2) A2: search reading to extract information on a predetermined topic (Search reading), (3) A3: identify specific details (Details), (4) A4: identify main ideas and significant points (Main ideas), (5) A5: make inferences (Inferences), (6) A6: understand the structure and cohesion of a passage (Logic), (7) A7: compare viewpoints (Viewpoints).

To evaluate the appropriateness of the above Q-matrices based on G-DINA, absolute fit statistics were used. As shown in Table 3, both Q-matrices could be accepted based on the *p* statistics with adjusted *p*-value > 0.05. However, previous research shows that the *p* statistics often exhibit low rejections, making them insensitive to Q-matrix misspecification (Chen et al., 2013). Q1 was rejected by both *r* (adjusted *p*-value < 0.01) and *l* (adjusted *p* < 0.05), suggesting likely misspecifications in the Q-matrix (Chen et al., 2013). In comparison, Q2 provided a good fit based on both *r* and *l* statistics (adjusted *p*-value > 0.05) and was submitted as a provisional Q-matrix for further validation.

**Table 3:** Absolute model fit indices for Q1 and Q2.

Q- matrix	Max. z(*p*)	*p*-value	adj. *p*-value	Max. z(*r*)	*p*-value	adj. *p*-value	Max. z(*l*)	*p*-value	adj. *p*-value
Q1	0.1768	0.8597	1	4.3282	0.0000	0.0070	4.0282	0.0001	0.0261
Q2	0.1990	0.8423	1	3.0992	0.0019	0.9022	3.1754	0.0015	0.6958

adj. *p*-values were based on the Holm method. Max. z(*p*), Max. z(*r*) & Max. z(*l*) refer to maximum z-score for *p, r* and *l*.

#### 4.1.4 Construction and validation of the final Q-matrix

Given that Q2 was accepted based on absolute fit evaluation, it was warranted to further refine Q2 at the item level. The stepwise Wald test method (Ma and de la Torre, 2019b) was employed and the PVAF was set to 0.95 as the default. As a result, six entries across the 31 items were recommended for modification, as shown in Table 4. Since statistical recommendations from the Wald test could only be ancillary rather than decisive, a modification was implemented only when it was supported by converging evidence from expert judgments, students' verbalizations, and close inspection of the textual and cognitive demands of the item. Otherwise, the original specification was retained.

**Table 4:** Suggested and final Q-matrices.

Item	Suggested Q-matrix							Q3						
	A1	A2	A3	A4	A5	A6	A7	A1	A2	A3	A4	A5	A6	A7
1	0^*^	0	1^*^	0	0	0	0	1	0	0	0	0	0	0
2	0	1	0	0	0	0	0	0	1	0	0	0	0	0
3	0	1	0	0	0	0	0	0	1	0	0	0	0	0
4	0	1^*^	0	0	0	0	0	0	1	0	0	0	0	0
5	0	1	0	0	0	0	0	0	1	0	0	0	0	0
6	1	0	0	0	0	0	0	1	0	0	0	0	0	0
7	0	1	0	0	0	0	0	0	1	0	0	0	0	0
8	1	0	0	0	0	0	0	1	0	0	0	0	0	0
9	1	0	0	0	0	0	0	1	0	0	0	0	0	0
11	0	0	1	0	0	0	0	0	0	1	0	0	0	0
12	0	0	0	0	1	0	0	0	0	0	0	1	0	0
13	0	0	1	0	0^*^	0	0	0	0	1	0	1	0	0
14	0	0	1	1	0	0	0	0	0	1	1	0	0	0
15	0	0	1	0	0	0	0	0	0	1	0	0	0	0
16	0	0	0	1	0	0	0	0	0	0	1	0	0	0
17	0	0	1	0	0	0	0	0	0	1	0	0	0	0
18	0	0	0	0	1	0	0	0	0	0	0	1	0	0
19	0	0	1	0	0	0	0	0	0	1	0	0	0	0
20	0	0	1	0	0^*^	0	0	0	0	1	0	0	0	0
21	0	0	1^*^	0	0^*^	0	0	0	0	0	0	1	0	0
22	0	0	0	1	0	0	0	0	0	0	1	0	0	0
23	0	0	0	1	0	0	1	0	0	0	1	0	0	1
24	0	0	0	1	0	0	1	0	0	0	1	0	0	1
25	0	0	0	0^*^	0	0	1	0	0	0	1	0	0	1
26	0	0	0	1	0	0	1	0	0	0	1	0	0	1
27	0	0	0	1	0	0	1	0	0	0	1	0	0	1
28	0	0	0	1	0	1	0	0	0	0	1	0	1	0
29	0	0	0	1	0	1	0	0	0	0	1	0	1	0
30	0	0	0	1	0	1	0	0	0	0	1	0	1	0
31	0	0	0	1	0	1	0	0	0	0	1	0	1	0
32	0	0	0	1	0	1	0	0	0	0	1	0	1	0

^*^denotes a modified element.

The model suggested item 1 measured the careful reading skill of A3. In the UDig test, time constraint and item types in the expeditious reading section clearly factored into students' strategy selection as they approached the text and test items. In this vein, the study distinguished expeditious reading strategies from careful reading strategies on an operational level. Hence, item 1 was not revised. Item 4 was reassigned to Search reading from Scanning based on expert judgments (50% agreement) and textual evidence requiring multi-sentence syntactic processing other than simple word matching. Items 13, 21, and 25 remained unmodified given consensus between expert judgments and students' verbalizations. As to item 20, Inference (A5) was included in Q2 as two experts specified it as a primary subskill. Item 20 asked students to select a statement about the World Marathon Majors series. The key was “It is sometimes difficult to get an entry into the races.” The relevant information in the reading passage states that “…even getting to the start line is a challenge in itself. All five races regularly sell out, with London, New York and Boston massively oversubscribed…” In this case, textual evidence (e.g., “getting to the start line is a challenge,” “regularly sell out,” “oversubscribed”) clearly reflected the difficulty to get into the race and readers did not need to go beyond explicit ideas and use background knowledge to make predictions or hypothesis. Therefore, inferencing was deleted from the Q-matrix. In sum, two modifications were made based on the suggestions and a final Q-matrix (Q3) was constructed consequently (see Table 4). As Table 5 shows, absolute fit evaluation of Q3 also manifested satisfactory model fit (adjusted *p*-value > 0.05 on the *p, r* and *l* statistics). Attribute-level classification accuracy (Pa) was reported as evidence of classification reliability. The estimated Pa values were 0.91 for Scanning (A1), 0.92 for Search reading (A2), 0.88 for Details (A3), 0.88 for Main ideas (A4), 0.96 for Inferences (A5), 0.96 for Logic (A6), and 0.89 for Viewpoints (A7). These results indicate high classification accuracy across all seven reading attributes. In addition, Table 6 presents the tetrachoric correlations among the seven attributes, which ranged from −0.02 to 0.76. Most attribute pairs showed weak to moderate correlations, whereas stronger correlations were observed between conceptually related attributes, including Scanning (A1) and Search reading (A2) (*r* = 0.76).

**Table 5:** Absolute model fit indices for Q3.

Q-matrix	Max. z(*p*)	*p*-value	adj. *p*-value	Max. z(*r*)	*p*-value	adj. *p*-value	Max. z(*l*)	*p*-value	adj. *p*-value
Q3	0.1564	0.8757	1	3.1912	0.0014	0.6588	3.2713	0.0011	0.4978

adj. *p*-values were based on the Holm method. Max. z(*p*), Max. z(*r*) & Max. z(*l*) refer to maximum z-score for *p,r and l*.

**Table 6:** Tetrachoric correlations among the attributes.

Attribute	A1	A2	A3	A4	A5	A6	A7
A1	1						
A2	0.76	1					
A3	0.69	0.40	1				
A4	0.27	0.21	0.71	1			
A5	0.04	−0.02	0.24	0.25	1		
A6	0	0.10	0.01	0.03	0.20	1	
A7	0.34	0.17	0.51	0.30	0.07	0.11	1

### 4.2 Characteristics of test-takers' reading subskill mastery

#### 4.2.1 Selection of the best – fitting model

Given that a validated Q3 had been obtained on the basis of a saturated model, relative fit statistics to compare G-DINA with rival reduced models including DINA, DINO, A-CDM, and R-RUM were examined. Smaller value for each of the three statistics stood for a better fit. Table 7 presents the relative fit indices of the deviance, AIC, and BIC. As can be seen, DINO had the worst fit across all three statistics, followed by DINA. DINO is compensatory: mastering any single required skill gives an examinee the same probability of a correct response as someone who has mastered all the skills. In contrast, DINA is a non-compensatory model, requiring all specified skills to be mastered to arrive at the correct answer for an item. Thus, both models may be overly restrictive for assessing reading comprehension. As to A-CDM and R-RUM, the values of BIC were slightly smaller than G-DINA. This occurs because BIC tends to heavily penalize models with greater complexity (Li et al., 2016). However, compared to the small decrease in the values of BIC, the increase in the values of deviance and AIC of A-CDM and R-RUM were more substantial. Taken as a whole, G-DINA provided the best fit relative to the other four models. Moreover, unlike the reduced CDMs, G-DINA does not impose a single condensation rule across items. Instead, it allows different items to combine attributes in either compensatory or non-compensatory ways, providing greater flexibility for modeling the complex cognitive processes involved in reading comprehension (Ravand, 2016; Ravand et al., 2024). The item-level model selection results based on the Wald test are summarized in Table 8. As can be seen, different condensation patterns were observed across task types. Most selected-response items (multiple-choice and summary completion) were best fit by G-DINA, whereas the cross-text multiple matching task was represented by a mixture of compensatory and non-compensatory reduced CDMs, indicating that attribute interactions varied across items and could not be characterized by a single processing rule. By contrast, most gapped-text items were represented by LLM, suggesting a relatively consistent additive interaction between Main ideas (A4) and Logic (A6). This pattern may reflect the cognitive demands of the gapped-text task, which require students to understand the development of ideas across the whole text while evaluating the logical and cohesive relationships surrounding each gap. Thus, Main ideas (A4) and Logic (A6) may make independent contributions to successful task performance: understanding the overall structure helps readers anticipate what type of information is required at a given position, whereas recognizing logical connections helps them evaluate whether a candidate sentence fits with the surrounding text. This finding also highlights how task characteristics may shape the ways in which reading attributes interact. Overall, the results further support the choice of G-DINA, as different reading tasks appear to involve different patterns of attribute interaction rather than a uniform condensation rule.

**Table 7:** Relative model fit indices.

Model	Deviance	AIC	BIC
G-DINA	22,425.96	22,851.96	23,802.92
DINA	22,598.65	22,976.65	23,820.46
DINO	22,630.19	23,008.19	23,852
A-CDM	22,489.73	22,891.73	23,789.11
R-RUM	22,483.91	22,885.91	23,783.29

**Table 8:** Item-level model selection based on the Wald test.

Task type	Items	Subskills	Selected model(s)
True/false	1–9	A1; A2	G-DINA
Multiple choice & summary completion	11–22	A3; A4; A5	Predominantly G-DINA (Item 13 R-RUM)
Cross-text multiple matching	23–27	A4; A7	Mixed (A-CDM; LLM; R-RUM; DINA)
Gapped text	28–32	A4; A6	Predominantly LLM (Item 28 G-DINA)

#### 4.2.2 Test-takers' strengths and weaknesses in reading subskills

**Figure 1:**
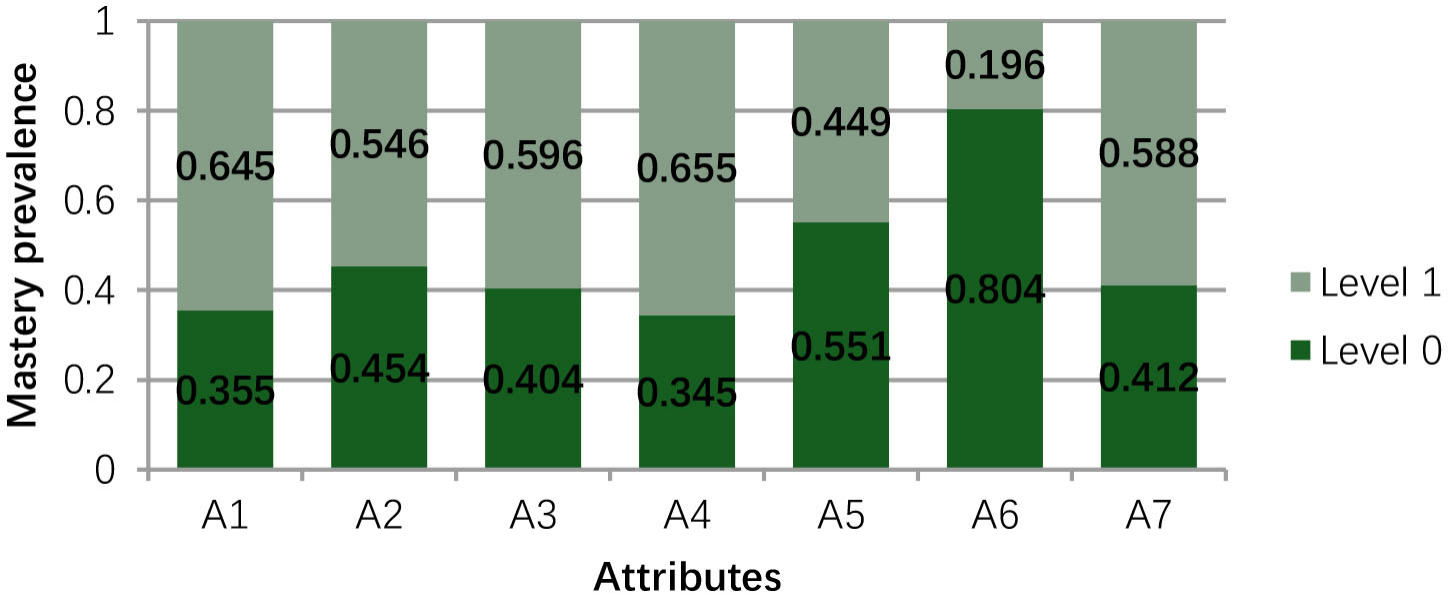
Attribute prevalence.

With the validated Q-matrix and the best-fitting model, individual mastery profiles were generated for all test-takers. In terms of the overall pattern of test-takers' reading subskills, Figure 1 shows the prevalence of each attribute, reflecting the sample-level probability of mastery and the relative difficulty of the attributes. In Figure 1, level 1 indicates the mastery of the subskill, while level 0 stands for non-mastery. Logic (A6) emerged as the most difficult attribute, mastered by only 19.6% of the test-takers. This is followed by Inference (A5), mastered by 44.9% of students. Conversely, Main ideas (A4) and Scanning (A1) emerged as the easiest attributes, mastered by 65.5% and 64.5% of the test-takers. Regarding the expeditious reading subskills, Search reading was found to be more difficult than Scanning, as expected.

Cognitive diagnostic modeling also categorized test-takers into different latent classes of mastery and non-mastery subskill patterns. With seven defined attributes in this study, 2^7^ (i.e., 128) latent classes or attribute patterns were the maximum possible patterns. Table 9 shows the estimated proportions of the first 10 latent classes which had the highest class probabilities. From Table 9, it was clear that the latent class “1111101” demonstrated the highest class probability (0.1067), indicating that the latent class of mastering all attributes except for Logic was the most prevalent one. This was in accordance with the results from attribute prevalence as Logic tended to be the most difficult subskill for students to master. The latent class “1111001” came second, indicating the difficulty of mastering Inference. Among the most prevalent latent classes, only the attribute patterns “11,” “10,” and “00” were observed for Scanning (A1) and Search reading (A2). Examination of the complete latent class distribution, however, showed that the “01” pattern was present in a small proportion of respondents. Therefore, the results do not provide conclusive evidence for a prerequisite relationship between the two subskills.

**Table 9:** Latent class and posterior probabilities.

#	Latent class	Posterior probability
1	1111101	0.1067
2	1111001	0.0828
3	0000000	0.0530
4	1111000	0.0439
5	1011001	0.0393
6	1110001	0.0353
7	1100000	0.0348
8	0001000	0.0340
9	0011001	0.0318
10	1011101	0.0287

## 5 Discussion

This study was conducted to validate a purpose-built diagnostic assessment, namely the UDig Reading Test. Instead of only resorting to the test developer's initial intention, students' actual reading processes, expert judgments, and cognitive statistical modeling were triangulated to better understand the reading subskills being diagnosed. The empirical validation largely confirmed the proposed item-subskill associations, although several misspecifications were identified. It was found that the final Q-matrix based on empirical data outperformed the initial Q-matrix derived from test developer's specifications. The results indicate that a consensus on the item-by-subskill relationships is not easily achieved among the test developer's intention, expert judgments and students' verbalizations. As Alderson (2000) points out, although test developers may intend items to target certain subskills, whether they succeed may not much matter since reading scores are not reported by subskills. However, in diagnostic assessment, which claims to identify strengths and weaknesses in language subskills, inaccurately specified associations pose serious threats to the validity of the test. As the educational value of such assessment depends, to a large extent, on the accuracy of the diagnostic information it produces, misspecified item-subskill relationships may lead to misleading diagnostic feedback, potentially resulting in inappropriate instructional decisions and remedial support. Thus, it is of paramount importance to carefully specify and iteratively scrutinize the associations between test items and the constructs in the test construction and validation process. Since the UDig system was still in its trial stage, the conflicting evidence regarding the item-by-subskill relationships, which might jeopardize the validity of the UDig Reading Test, should be extensively reviewed and carefully modified by the test developer. By strengthening the validity of the Q-matrix and the precision of attribute-level diagnosis, the present study provides a stronger empirical foundation for diagnostic feedback and its subsequent use in supporting tailored teaching and learning.

A key finding of this study is the empirical specifications of cognitive subskills within the expeditious reading domain. Expeditious reading takes place when readers process texts quickly, selectively, and efficiently, whilst careful reading involves slow, deliberate, sequential, and incremental processing of the text (Khalifa and Weir, 2009; Urquhart and Weir, 1998). In the UDig Reading Test, both scanning and search reading serve as representative expeditious reading strategies, associated with the true or false item type. Scanning is typically achieved through exact word matching and the process mostly takes place at the local level, involving the decoding of words and little or no syntactic processing. Compared to scanning which relies on simple visual matching, search reading requires readers to stay alert to semantically related terms and pay closer attention to the text to extract embedded information (Pugh, 1978). Analysis of students' verbalizations showed that search reading posed a significant challenge for students, corroborating Jin's (1998) finding in the introspection study. Students frequently resorted to simple word-matching strategies between the test items and the text, and had difficulty in integrating relevant information and processing connections or transitions. Moreover, distinguishing between scanning and search reading proved challenging not only for students but also for expert raters, who demonstrated lower confidence and consistency in assigning these two strategies. Interestingly, although scanning and search reading showed a relatively strong correlation (*r* = 0.76), both attributes also demonstrated high classification accuracy. This pattern suggests that the two expeditious reading strategies share overlapping cognitive processes characterized by rapid, selective, and goal-directed information location, while remaining empirically distinguishable because they differ in the extent of semantic processing required. However, the boundary between the two strategies may be less discrete than suggested by theoretical definitions and may therefore be challenging to maintain in practice, as reflected in the lower agreement observed among expert raters. Nevertheless, such distinctions remain valuable in purpose-built diagnostic assessment, where the goal is to provide fine-grained information about learners' strengths and weaknesses. Distinguishing between learners who rely primarily on surface lexical matching and those who can efficiently locate and process semantically relevant information may provide more informative evidence for subsequent instructional support. Taken together, the present findings highlight the need for greater emphasis on expeditious reading in both diagnostic assessment design and classroom instruction. A further observation concerns the mastery patterns of scanning and search reading. The absence of conclusive evidence for a hierarchical relationship between scanning and search reading has broader implications for understanding expeditious reading development. Rather than suggesting a strict linear developmental sequence, the present findings indicate that these closely related strategies may develop in a more flexible manner, with their deployment depending on the demands of particular reading tasks.

Beyond the expeditious reading domain, Logic (understanding the structure and cohesion of a passage) and Inferences (making inferences) emerged as the most challenging subskills for students. This pattern highlights the importance of helping learners move beyond surface-level local decoding and develop greater semantic alertness to recognize argument transitions and thematic links. Such higher-level cognitive processes are essential for successful reading comprehension. Incorporating these subskills into diagnostic assessment can provide teachers and students with more fine-grained information. Such information can help teachers identify learners' specific reading weaknesses and tailor subsequent instruction accordingly, while also helping students engage in more focused self-regulated learning.

In addition to these pedagogical implications, the present findings also raise important considerations for diagnostic assessment design and validation. As discussed earlier, researchers have consistently cautioned that retrofitting tests designed for non-diagnostic purposes is by no means an adequate approach to diagnosing students' language ability. This study addressed this concern by applying a cognitive diagnostic approach to the conventional UDig test. While conventional diagnostic assessment has a clear diagnostic purpose to provide fine-grained subscores for stakeholders, the psychometric qualities of these subscores are often questioned (Tannenbaum, 2019). Furthermore, typically only one attribute is assigned to each item in traditional diagnostic assessment like the UDig, whereas both prior research and the present study reveal that test-takers may utilize multiple processing strategies simultaneously in solving some tasks. For example, in the UDig Test, the cross-text multiple matching task not only required test-takers to compare and analyze viewpoints across texts, but also relied on the ability to identify main ideas and significant points. Similarly, the gapped-text task in which test-takers were asked to insert sentences into the blanks of the passage without losing logical coherence, demanded complex processing of the lexical, grammatical, and logical relationships, necessitating whole-passage comprehension. These findings indicate that higher-level subskills such as logical processing, rarely operate in isolation but instead interact with other cognitive processes during task completion. In this respect, the cognitive diagnostic approach, which accommodates multiple attributes assigned to one test item and models interactions between these attributes, aligns more closely with the actual reading process. The satisfactory model-fit indices obtained in this study further demonstrated the robustness of the CDMs in estimating students' reading subskill mastery probabilities, suggesting that the cognitive diagnostic approach could enhance conventional diagnostic tests by advancing diagnostic precision.

More broadly, the successful application points to the potential for developing cognitive diagnostic assessment batteries—diagnostic assessment in a form of cognitive psychometric diagnosis—from the outset. Within a CDA framework, a Q-matrix based on domain theories and empirical data is predetermined and validated. The best-fitting CDM(s) are automatically selected and performed to generate mastery profiles, and well-designed automated feedback reports are delivered to stakeholders. As Alderson et al. (2014) put it, up to now only a few tests have been designed to provide diagnostic information and even these tests only “represent an impoverished view of what diagnostic assessment is capable of” (p. 237). The development of CDA therefore holds great potential for shifting assessment paradigm from macro-level performance classification toward more micro-level cognitive diagnosis and tailored learning support.

## 6 Conclusion

This study is a response to the overdue call for research into purpose-built diagnostic assessment. Through applying a cognitive diagnostic approach, various types of evidence were accumulated to validate the UDig Reading Test. In the validation process, threats to the test's validity were pinpointed, and recommendations for revisions were proposed. In this way, the diagnostic reading assessment itself has been effectively diagnosed. Cognitive diagnostic modeling is a burgeoning field but its application in language assessments remains inadequate. The study followed a rigorous procedure to apply cognitive diagnostic modeling, which can serve as a practical framework for future studies aiming to adopt this robust approach. One limitation of this study is the lack of investigation into the use or impact of the UDig feedback. The potential of diagnostic assessment to enhance learning hinges not only on the accuracy of the diagnostic information it provides, but also on whether diagnostic feedback is communicated clearly and in a timely manner, enabling teachers and students to make informed decisions. Therefore, future studies should investigate how diagnostic feedback is communicated, interpreted, and acted upon by teachers and students across diverse educational contexts. Such questions may be most effectively addressed through longitudinal classroom-based intervention studies, complemented by qualitative approaches (e.g., classroom observations, reflective tasks, and interviews) to document the real-world utilization of diagnostic feedback, examine the specific actions test users take, and evaluate the impact of remediation on teaching and learning. It is hoped that by devoting more research to the field and fostering more collaboration among test developers, researchers, and test users, the capacity of diagnostic assessment to inform instruction and learning can be fully realized.

## Data Availability

The datasets presented in this article are not readily available because they are subject to confidentiality agreements related to the assessment materials. Requests to access the datasets should be directed to Hang Sun, irishangsun@usst.edu.cn.
